# Transcranial Direct Current Stimulation Optimization – From Physics-Based Computer Simulations to High-Fidelity Head Phantom Fabrication and Measurements

**DOI:** 10.3389/fnhum.2019.00388

**Published:** 2019-10-31

**Authors:** Leon Morales-Quezada, Mirret M. El-Hagrassy, Beatriz Costa, R. Andy McKinley, Pengcheng Lv, Felipe Fregni

**Affiliations:** ^1^Department of Physical Medicine and Rehabilitation, Neuromodulation Center, Spaulding Rehabilitation Hospital, Harvard Medical School, Boston, MA, United States; ^2^Air Force Research Laboratory, United States Air Force, Wright-Patterson AFB, Dayton, OH, United States; ^3^Alphasense, Wilmington, DE, United States

**Keywords:** transcranial direct current simulation, electric stimulation, electric conductivity, computer simulations, anatomic models, EEG, head phantom model, feasibility study

## Abstract

**Background:**

Transcranial direct current stimulation (tDCS) modulates neural networks. Computer simulations, while used to identify how currents behave within tissues of different conductivity properties, still need to be complemented by physical models.

**Objective/Hypothesis:**

To better understand tDCS effects on biology-mimicking tissues by developing and testing the feasibility of a high-fidelity 3D head phantom model that has sensing capabilities at different compartmental levels.

**Methods:**

Models obtained from MRI images generated 3D printed molds. Agar phantoms were fabricated, and 18 monitoring electrodes were placed on specific phantom brain areas.

**Results:**

When using rectangular electrodes, the measured and simulated voltages at the monitoring electrodes agreed reasonably well, except at excitation locations. The electric field distribution in different phantom layers appeared better confined with circular electrodes compared to rectangular electrodes.

**Conclusion:**

The high-fidelity 3D head model was found to be feasible and comparable with computer-based electrical simulations, with high correlation between simulated and measured brain voltages. This feasibility study supports testing to further assess the reliability of this model.

## Introduction

Transcranial direct current stimulation (tDCS) has been studied for decades and has potential therapeutic effects for a wide range of medical conditions, as well as for cognitive enhancement in healthy individuals (Andy McKinley et al., 2013). With the tDCS method, weak currents (e.g., 2 mA) are injected through the scalp, which then modulates neural activity in a polarity-dependent fashion at the targeted symptom- or task-specific brain areas/networks to enhance function. Although tDCS holds great promise, it is not straightforward to determine optimum treatment procedures due to the complex shapes/configurations and the dramatic conductivity differences among various tissues, including the scalp, skull, cerebrospinal fluid (CSF), gray matter, etc. Generally, placing the stimulating electrodes directly over targeted brain areas cannot guarantee the modulation of those areas, although this assumption is typically held when designing stimulation protocols. In addition to stimulating (active) electrode placement in target locations, other therapeutic treatment parameters need to be optimized, including the amplitude of the injection current, the number of injection electrodes, the surface areas of the active and reference electrodes and their configurations/montage. The use of computer simulations is one approach to improve therapeutic parameters.

Computer simulations have helped identify how currents behave over recent years (Schmidt et al., 2015); while useful, they have many limitations. For example, they make assumptions for tissue conductivities, but different assumed values can lead to highly different results in electric field magnitudes (Laakso et al., 2015; Saturnino et al., 2015). Other factors altering electric fields include registration procedure errors, anatomic variations (Laakso et al., 2016), functional connectivity and inter-individual variability. It is thus important to investigate current flow in a structural model reflecting human brain macroanatomy and its different anatomical compartment conductivities in order to further validate such a model in human participants. Animal studies do not translate current distribution in human brains, although two studies on transcranial alternating current stimulation (tACS) in epilepsy patients with invasive recordings (one also included Cebus monkeys) (Opitz et al., 2016) found different degrees of voltage attenuation at higher frequencies (Opitz et al., 2016; Huang et al., 2017), and that measured voltages were comparable to predictions (Huang et al., 2017). Meanwhile, postmortem electric stimulation attempts have been limited by live and dead tissue differences (Vöröslakos et al., 2018); studying the brains of deceased patients who had deep brain stimulation (Pilitsis et al., 2008; Reddy and Lozano, 2018) could not directly validate electric current paths nor their effects on living tissues. However, previous studies using real-skull phantom heads reliably registered modeled electroencephalography (EEG) sources (Leahy et al., 1998; Baillet et al., 2001). Similarly, materials with different conductivity (e.g., ceramics, clay) (Hunold et al., 2018) have been used to model human skull geometry in realistic transcranial electrical stimulation head phantoms, and initial attempts to apply image-guided tDCS based on phantom modeling successfully increased the electric current to pre-defined target areas (Jung et al., 2013; Kim et al., 2015).

Head models should be realistic and account for anatomical variability to better understand data generated by them; in that way, accurate measurement methods on phantom heads will help estimate *in vivo* current diffusion throughout different tissue conductivities. Additionally, 3D head phantom models can measure currents in real time and be used for training in different settings, e.g., controlled laboratory or uncontrolled clinical environments.

**FIGURE 1:**
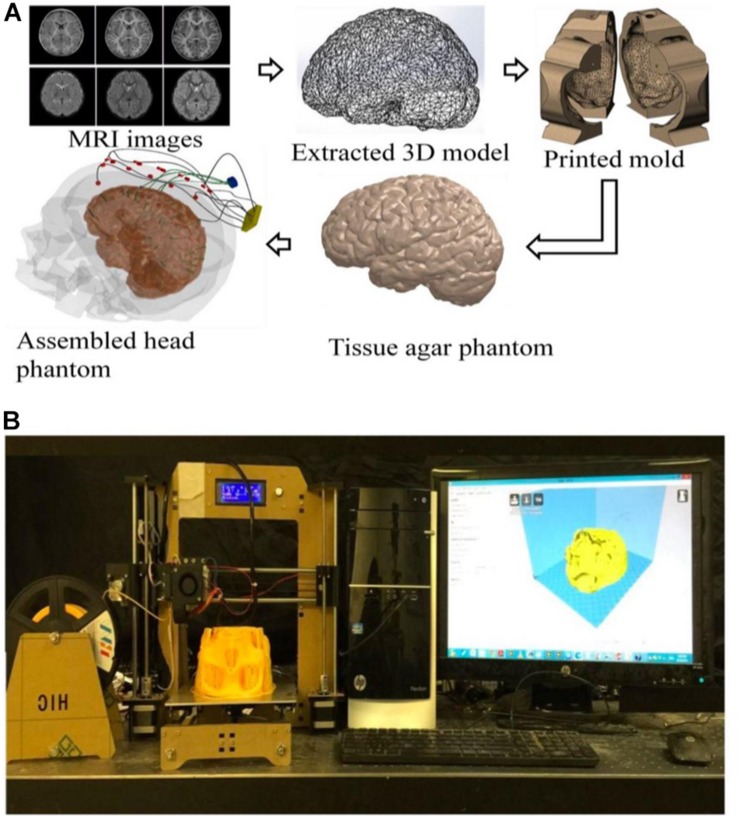
**(A)** An illustration of the process flow to fabricate a high-fidelity head phantom for tDCS model validation; **(B)** a picture of the 3D filament-based 3D printer for tissue mold printing.

This research presents an innovative method to implement a high-fidelity head phantom for tDCS optimization (Figure 1). Initial feasibility data were collected from the implemented head phantom and compared with theoretical data obtained using computer simulations (Supplementary Figures S1, S2). The objective of our phantom model was to review and analyze the theoretical results obtained from physics-based modeling/simulations. In our model, while different tissue conductivities were assumed, yet sensing electrodes in some anatomical compartments allowed us to observe differences among tissues. We developed a high-fidelity 3D head phantom model to replicate anatomical features, allowing us to more accurately measure electric current activity at different head compartments and thereby determine optimum tDCS therapeutic parameters. We provide detailed information on the 3D head phantom fabrication process as well as initial currents measurements data obtained from this phantom.

## Materials and Methods

### Extraction of 3D Models for Different Tissues From Head MRI Image Stack

The models used in this paper were extracted from Subject #18’s magnetic resonance imaging (MRI) (Figure 1) in the open-source Simulated Brain Database (McGill University, Montreal, Canada) (Baillet et al., 2001; Aubert-Broche et al., 2006; BrainWeb, 2019) using the following two steps: (a) 3D point cloud extraction and (b) post processing of the obtained 3D point clouds and surface triangulation of the individual tissue model.

**FIGURE 2:**
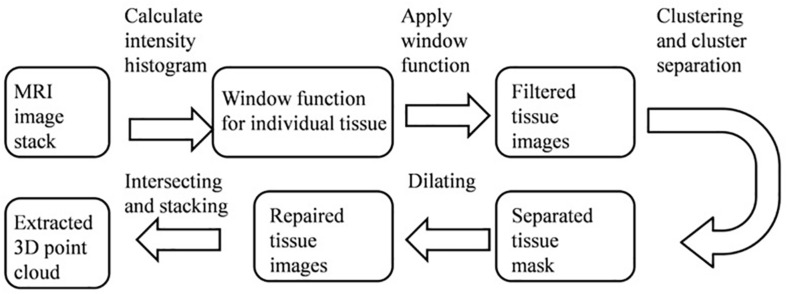
A block diagram showing the steps to extract the 3D point clouds of individual tissue from an MRI image stack.

Figure 2 shows a block diagram of the extraction stage: obtaining the 3D point clouds for different tissues based on an MRI image stack. Firstly, a histogram of the gray-scale intensity of individual pixels in an image stack was obtained. Different tissues (e.g., skull, gray, and white matter) have different gray-scale intensities, so a window function/band pass filter (determined by a Gaussian fitting of such a histogram) was first applied to the raw MRI image to roughly separate a tissue from its surroundings. A cluster opening algorithm was subsequently applied to find the largest connected cluster, which can further separate such a tissue from its neighboring tissues. Due to the cluster opening, certain areas of the image appeared discontinuous or empty. Therefore, a dilating step was used to grow the separated tissue and retrieve points lost during the cluster opening process. The resulted repaired image after the dilation step was slightly larger than the actual tissue. Finally, a Boolean operation (i.e., intersection) was used to extract common regions between the dilated and the filtered images to completely reveal the data points of a tissue contained in an MRI image. Consequently, a complete set of 3D point cloud of a tissue can be obtained by repeating the same processes for each MRI image in a stack and piling those extracted data points along the height direction. It should be noted that the above-mentioned image segmentation steps have been widely used for processing of MRI and Computed Tomography (CT) images. Alternatively, one can also obtain the segmented images and meshed tissue models using commercial 3D image processing software [e.g., Simpleware ScanIP (3D Image, 2019; Basic Segmentation, 2019)], which employs similar processing techniques.

**FIGURE 3:**
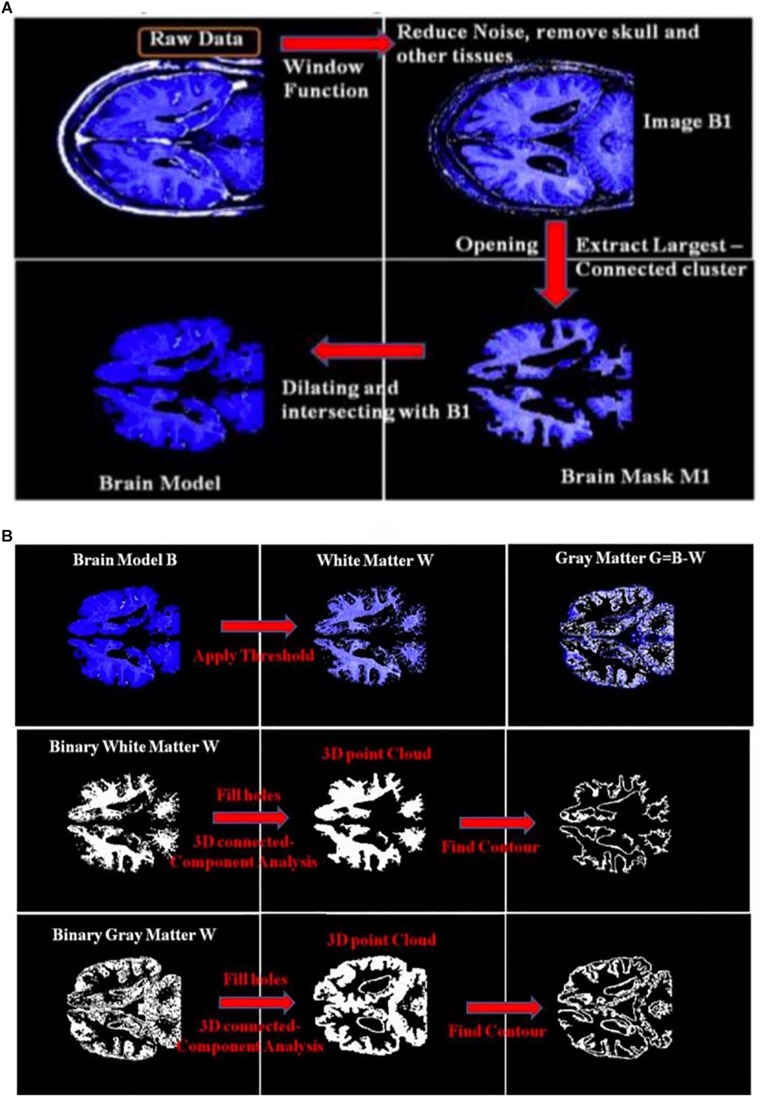
**(A)** An illustration of the process of obtaining a brain model using an MRI image; **(B)** an illustration of the process to obtain the 3D point clouds and surface contours of the white matter and gray matter from the extracted brain model. B1, Background Image 1; M1, Brain Mask 1.

**FIGURE 4:**
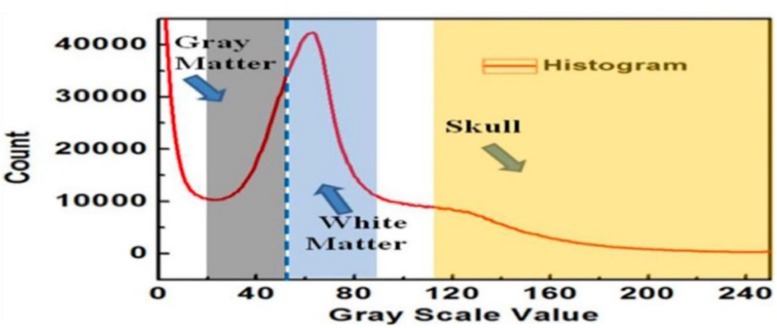
The histogram of the MRI image stack and different window functions applied to separate gray matter, white matter, and skull.

Figure 3A shows an example of extracting the brain data points from an image within an MRI image stack using the above-mentioned steps. The histogram of pixel gray-scale intensity values within such a stack is shown in Figure 4. Based on the histogram, window functions/band pass filters for different tissues were determined by Gaussian fittings of multiple peaks (i.e., 3 in the current case, which, respectively, corresponded to the white matter, gray matter, and skull) and shown as the shaded regions in the figure. For example, the skull area in the image appeared to be brighter than other tissues, and its gray-scale intensity level was found to be greater than ∼108. By applying a low pass filter (e.g., gray-intensity level lower than 88), one can roughly separate the brain from the skull (i.e., background image B1 in– Figure 3A). The brain tissue mask (i.e., background image in Figure 3A) was further separated using a clustering and cluster separation step (necessitated by overlapping gray-scale intensity values between brain and skull). Finally, the brain data points were extracted by dilating the brain tissue mask M1 and intersecting the resultant image with the filtered background image B1.

Figure 3B illustrates the process to obtain the 3D point clouds and surface contours for the gray matter and white matter from the extracted brain image. Data points that belong to the white matter (W) were first obtained by applying its corresponding window function/band pass filter to the extracted brain image. The gray matter (G) was then obtained by subtracting the white matter (W) from the brain image (B). The separated images of the white matter and gray matter were converted into binary images by setting proper gray-scale intensity thresholds obtained from the histogram in Figure 4. Empty regions were filled, and algorithms were applied to identify the largest connected clusters in each of the white- and gray-matter binary image. By stacking those binary images along the depth direction of the MRI image stack, the 3D point clouds for the white and gray matter were obtained. Finally, those 3D point clouds were further processed using open-source software MeshLab (Cignoni et al., 2008; Laakso et al., 2016) to remove redundant data points and to obtain the surface mesh structures by performing surface triangulations. Consequently, stereolithography (.stl) files containing the 3D information of brain tissues (describing the surface structures of gray matter and white matter) were generated.

**FIGURE 5:**
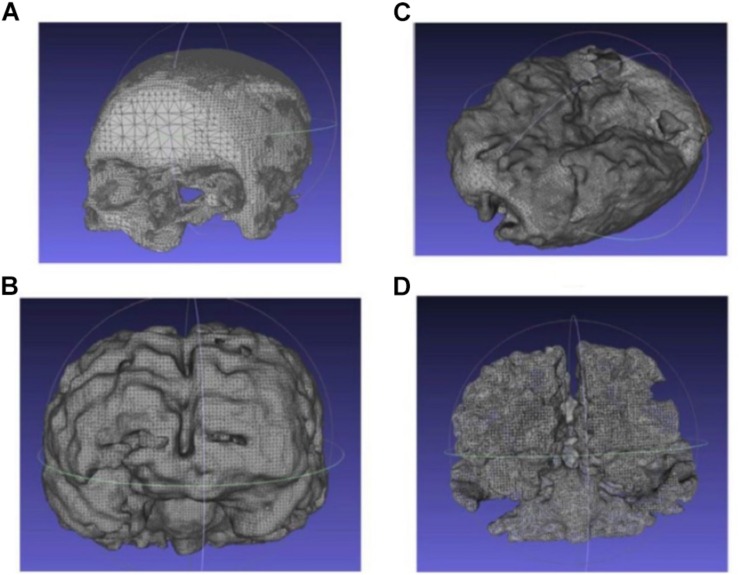
Extracted and processed tissue models from the MRI image stack **(A)** skull, **(B)** CSF, **(C)** gray matter, and **(D)** white matter.

Similarly, the 3D point clouds and .stl files for other tissues were obtained using the above-mentioned methods. Figure 5 shows the obtained .stl files for the skull, CSF, gray matter and white matter, respectively. The skin/scalp model (not shown in the picture) was obtained by dilating the skull model outward by 8 mm and then subtracting the skull from the dilated model.

### 3D Printed Molds and Agar Phantom Fabrication for Different Head Tissues Using 3D Printing

**FIGURE 6:**
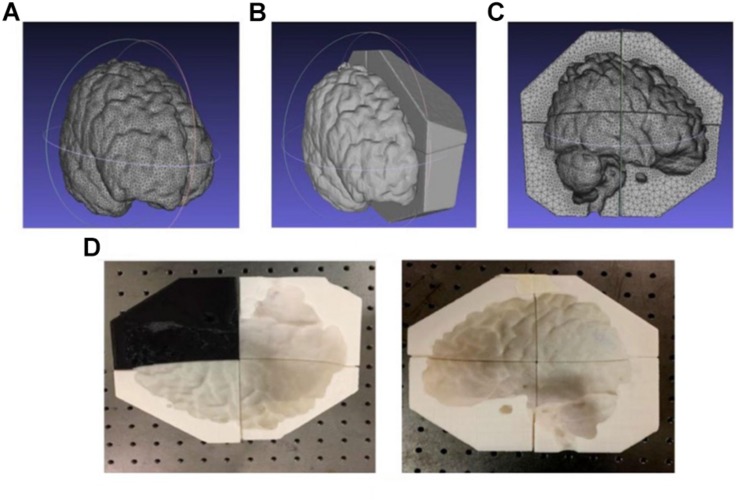
**(A–C)** The .stl files for the gray matter and **(D)** printed mold pieces.

The scalp, skull, and gray matter (but not white matter) 3D models (in *.stl* files) were extracted and analyzed as described above and subsequently enclosed by larger volumes using Solidworks (SolidWorks, Dassault Systems, Vélizy-Villacoublay, France). After a Boolean subtraction, we obtained mold cavities with shape and configuration complementary to individual tissues and segmented each mold into 6–8 pieces (depending on size and complexity) to facilitate assembly and disassembly. Such tissue mold files are subsequently sent to a commercial filament-based Fused Deposition Manufacturing (FDM) 3D printer (HICTOP Prusa i3) for fabrication. All the cavity molds and shell model (discussed below) were fabricated using this 3D printer. Figures 6A–C show the process of generating the mold model of gray matter for subsequent 3D printing, as discussed above. The .stl file of the gray matter was first enclosed in an irregular prism. In principle, a rectangular box or a cube can be used to enclose the gray matter. However, an irregular prism was used instead to minimize the overall volume of the mold pieces while retaining a satisfactory mechanical strength and structural stability for subsequent agar molding. Consequently, the tissue molds can be fabricated with significantly reduced time and filament material. Figure 6D shows the fabricated 3D printed mold for the gray matter containing 8 pieces for subsequent agar molding and disassembling.

White matter’s complex shape and configuration precludes harvesting a phantom in the same fashion (i.e., it is not possible to disassemble mold pieces in the same fashion as the simpler gray matter). Therefore, instead of forming a shape-complementary mold cavity, we used the extracted white matter tissue model to 3D print a hollow shell of the white matter. We then drilled tens of holes (with 6 mm diameters) at random locations on the white matter shell to allow subsequent agar molding and electrical conduction among white and gray matter phantoms.

**FIGURE 7:**
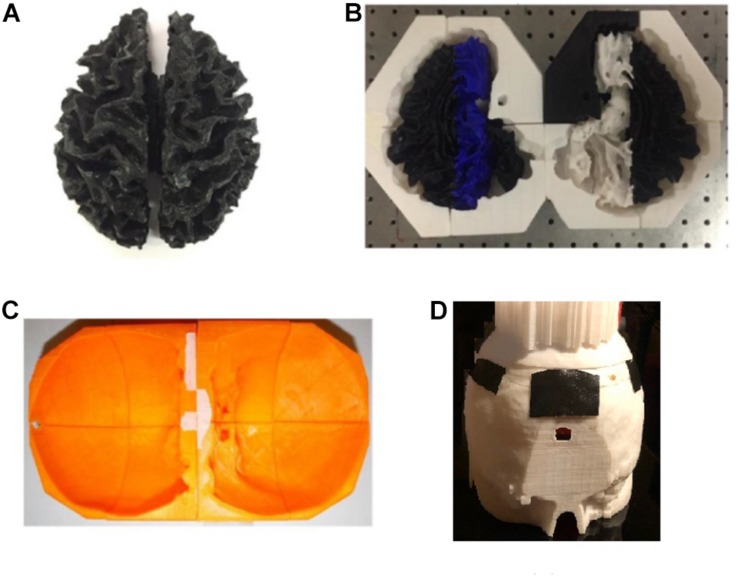
**(A)** white matter agar phantom shell, **(B)** white matter (with grooves) enclosed by the gray matter mold, **(C)** skull and **(D)** skin/scalp mold pieces.

**FIGURE 8:**
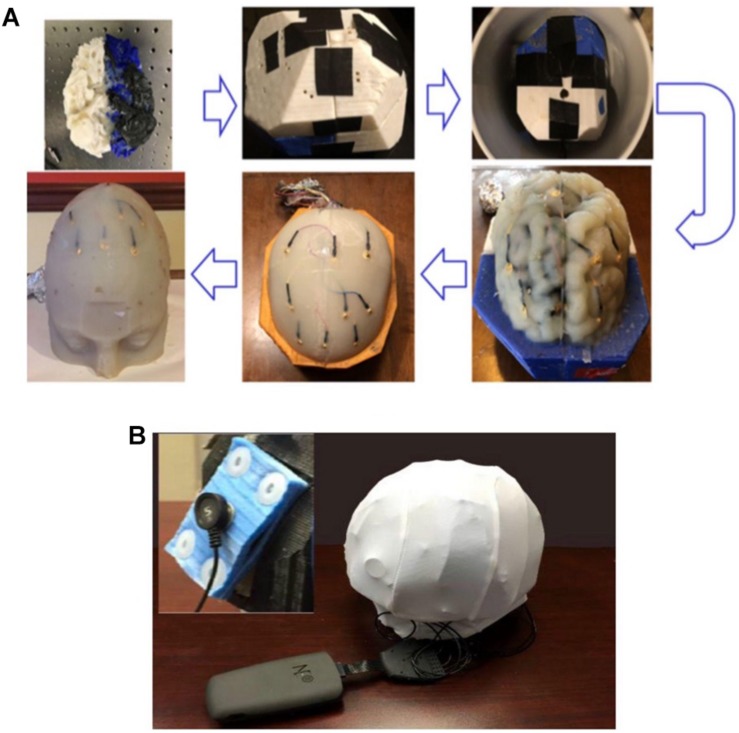
**(A)** The process flow to fabricate the four-layer head phantom^∗^ with embedded monitoring electrodes in the gray matter and skull layers; **(B)** a picture of the experimental setup to collect electrical responses with inset showing the rectangular tDCS electrode. ^∗^Color differences are due to the different filaments used to fabricate the mold and have no impact on conductivity.

Agar solutions with proper salt concentrations to simulate the electrical properties of various tissues were poured into their corresponding molds. This was done in the following way: based on the conductivities and compositions of the electrical simulants for different tissues as shown in Supplementary Table S1 (Bennett, 2011; Kandadai et al., 2012; Sperandio et al., 2012), a mixture of agar powder, sodium chloride and de-ionized water was prepared and heated to boiling temperature. Active agitation was used during the heating process to prevent the agar powders from agglomerating and settling to the bottom of the container. The heat was turned off after boiling and the agar solution was air-cooled at room temperature for 5 min. The partially gelated mixture was then poured into the 3D printed shell and molds and the whole assembly was placed in a refrigerator to allow complete cooling of the agar solutions. After the agar solutions were cooled down, the mold pieces were separated and removed to release the agar phantoms for different tissues. Figure 7 shows the white matter mold as well as the white matter enclosed by gray matter mold (further discussed below), skull and skin/scalp mold pieces. Figure 8A shows the sequence of harvesting individual tissue agar phantom and assembling the final head phantom process. As mentioned above, the 3D printed shell for white matter – which had been drilled with many 6 mm diameter holes – was immersed in the corresponding electrical simulant agar solutions. After the agar solution was cooled down, the white matter piece phantom was obtained by removing extra agar at different locations, such as those at the deep grooves of the white matter plastic model. The white matter agar phantom (with its grooves) was then embedded in the plastic mold of the gray matter, as shown in Figures 7B, 8A. Similarly, agar was poured into the mold and the phantom was harvested by separating the mold pieces after the agar cooled down.

**FIGURE 9:**
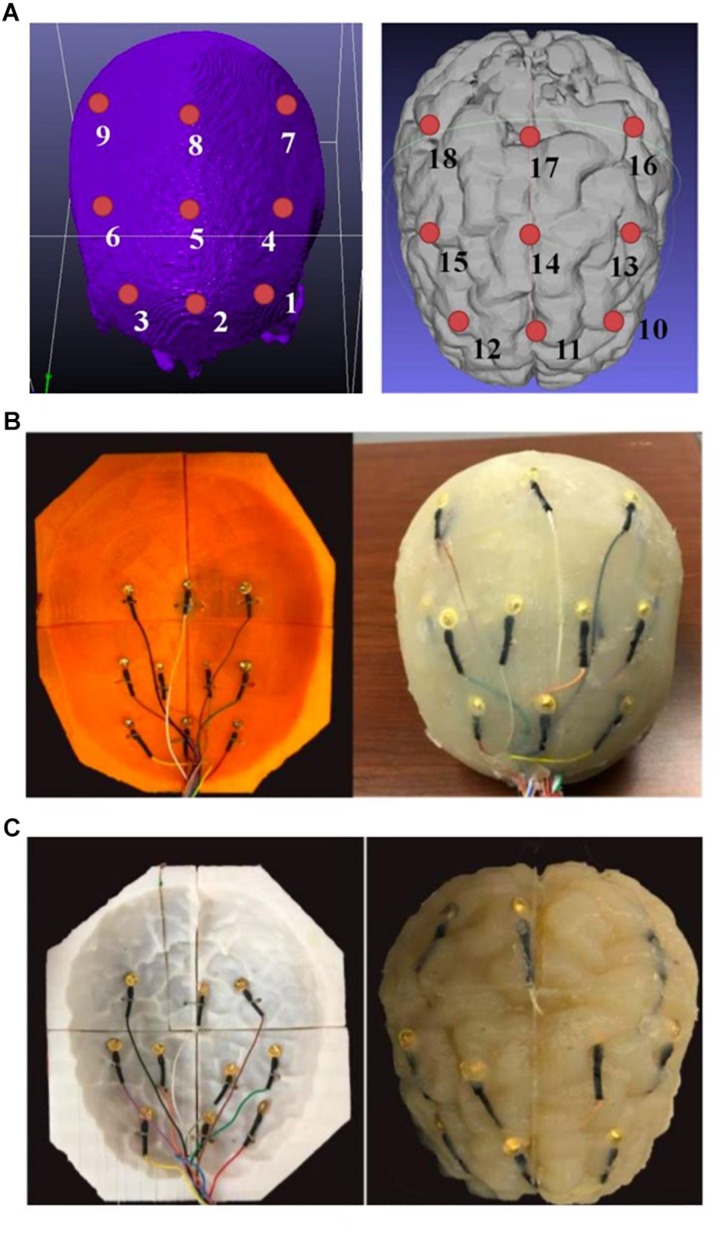
**(A)** A schematic of the positions of the monitoring electrodes on the skull (left) and gray matter tissue (right) layers; **(B)** embedding monitoring electrodes at the skull layer, and **(C)** embedding monitoring electrodes at the gray matter layer of the phantom.

During the agar phantom molding process, nine monitoring gold cup EEG electrodes were also embedded in the desired brain areas on the skull and gray matter tissue layers, respectively (Figures 8A, 9). In order to embed the monitoring electrodes at different tissue layers, small holes with ∼1 mm diameters were drilled at the desired locations of 3D printed molds, and during the agar molding process thin ribbon cables were used to loop around those electrodes to secure them in their places. After the agar was cooled down, those ribbon cables/wires were then removed before disassembling the mold pieces to harvest the gray matter phantom, thus leaving the monitoring electrodes embedded in the desired mold locations.

We therefore obtained a complete head phantom structure containing the white matter, gray matter, skull and skin/scalp layers with nine electrodes embedded in each of the gray matter and skull components. Figure 9A shows schematics of the relative positions of the nine monitoring electrodes at skull and the gray matter tissue layers, and Figures 9B,C shows pictures of the agar phantom with embedded electrodes in the gray matter and skull layers, respectively.

### Physics-Based tDCS Modeling

Theoretical responses under different tDCS stimulation conditions were studied using a commercial FEM simulation software, COMSOL MultiPhysics 4.1 (COMSOL, 2019). Individual tissue models extracted from the MRI image stack were assigned to an assembled 4-layer head phantom model consisting of white matter, gray matter, skull and skin/scalp layers. For physics-based modeling, the stimulation electrodes were placed on the skin layer using different montages (described in subsection “High-Speed Data Acquisition Circuit for Collection of Electrical Responses From Agar Phantom”). The effects of the monitoring electrodes embedded at different tissue layers should be negligible on the collected voltages. Therefore, those monitoring electrodes were not included in the FEM model to greatly simplify the model assembling processes and shorten computation time.

The tissue conductivities (in S/m) used for skin, skull, gray matter, white matter and stimulation electrode computations were 0.465, 0.01, 0.276, 0.126, and 5.99 × 10^7^, respectively. The continuity condition (*n● (J1 *−* J2)* = *0*) was assigned to all internal boundaries between tissues, and all external surfaces were treated as insulated. The boundary condition of inward current flow [i.e., *J* = *Jn* (normal current density)] was applied to the anode’s exposed surface, and ground was applied to the cathode’s exposed surface.

### 3D tDCS Experimental Data Collection and Model Simulation

After the physics and boundary conditions were set, the Laplace equation (∇● (σ∇V) = 0) was solved (Truong et al., 2013) to calculate the electrical potential and electrical field distribution at different tissues. The electrical responses under the anodal stimulation and return sources were simulated using both circular and rectangular electrodes. The circular electrodes were five electrodes of 2 cm diameters each, arranged evenly at the perimeter of a 5 cm circular support and embedded in circular custom-made sponges applied to the phantom. The rectangular electrodes were standard electrodes embedded in 35 cm^2^ rectangular sponges. Anode and cathode were of the same shape and size in each montage. The sizes of those electrodes were selected to be close to those used under actual tDCS treatment conditions, and they were placed over the skin layer. For tDCS model feasibility validation, the electrical responses under different stimulation conditions were acquired using the experimental setup shown in Figure 8B, which shows an example using the rectangular electrodes.

### High-Speed Data Acquisition Circuit for Collection of Electrical Responses From Agar Phantom

Supplementary Figure S1 shows the data acquisition device (DAQ) and a block diagram of the DAC for high-speed collections of electrical responses under different tDCS stimulation conditions. The amplitude of the stimulation current and pattern/montage of the stimulation electrodes can be varied using the DAQ device. Consequently, the collected electrical responses under different excitation patterns can be experimentally collected, and different tDCS current flow models can be verified.

A microcontroller was used to generate the control signals and collect the potential drops at the monitoring electrodes. The data acquisition process occurred as follows (Supplementary Figure S1a): Firstly, a set of montages and stimulation current (2 mA) was specified by the user in the graphical user interface (GUI) implemented in Labview. The corresponding commands were then sent from the computer to the microcontroller via a USB interface (Supplementary Figure S1b). After receiving the commands, the microcontroller generated the control signals/address bits accordingly to control a set of multiplexers (i.e., MUX). Consequently, the stimulation current from a 2 mA current source was sent to the corresponding stimulation electrode(s) placed in different tDCS montages. The montages were: (1) anode over the left primary motor cortex (PMC) and cathode over the right supraorbital region using each of the rectangular and circular electrodes for tDCS stimulation (but only the circular electrode montage was measured using monitoring electrodes); and another two montages that were both measured using recording electrodes: (2) anode over left dorsolateral prefrontal cortex (DLPFC), cathode over right DLPFC using rectangular electrodes; and (3) anode over left PMC and cathode over right PMC using circular electrodes.

Actual injection current in the phantom was monitored each time using a resistor in series connection with the injection electrode pair (anode and cathode). Actual current was tuned/adjusted via a voltage-controlled potentiostat to ensure that a fixed amount of current (2 mA in this study) was applied through the stimulation electrode pair throughout the whole measurement process. The potential drops on the 18 monitoring electrodes were recorded using another MUX device. The monitoring electrodes numbered 1 to 18 were sequentially swept by setting proper address bits on the MUX device. The voltage at each monitoring electrode was measured every 10 ms using the microcontroller’s analog-to-digital converter (ADC) channels. That is, 1 data point was taken every 10 ms, and 1 complete set of 18 data points took 0.18 s; the total data acquisition duration was 2 min, which led to more than 600 voltage data collected from each monitoring electrode. Such data were uploaded from the microcontroller to the computer and updated and displayed in real time for user visualizations. A median filter was also applied to the collected voltages to remove signal noises. The filtered data were then averaged to obtain a reliable potential reading under the stimulation condition.

## Results

**FIGURE 10:**
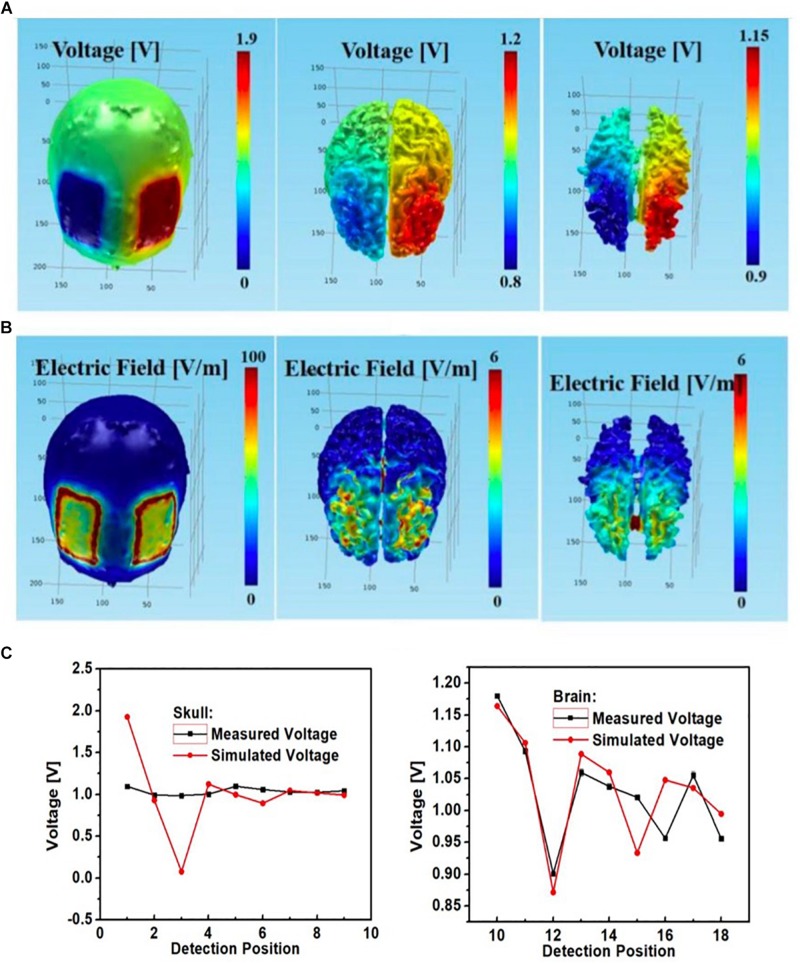
Bilateral DLPFC montage with anode over the left DLPFC, cathode over the right DLPFC using rectangular pads as the stimulation electrodes; simulated **(A)** electric potential and **(B)** electric field in skull (left), gray matter (middle) and white matter (right); **(C)** simulated and measured voltages at the nine monitoring electrodes in the skull (left) and brain (right) layers.

We were able to successfully develop a high-fidelity 3D head phantom model from MRI images and with sensing capabilities. The simulated montage using rectangular pad electrodes for bilateral DLPFC stimulation led to similar electric potential and field distributions in the gray matter and white matter (Figures 10A,B). Both the electric field and potential were much smaller in the gray matter and white matter layers than in the skull layer. Furthermore, there was agreement between simulated and measured voltages at the skull layer’s nine monitoring electrodes (Figure 10C), except at excitation locations (corresponding to EEG monitoring electrodes No. 1 and 3). Those had large discrepancies attributed to poor electric contact between the excitation electrodes and external skull surfaces. Voltages at the other skull electrodes agreed well. Measured voltages at the brain layer were close to simulated values (Figure 10C right).

**FIGURE 11:**
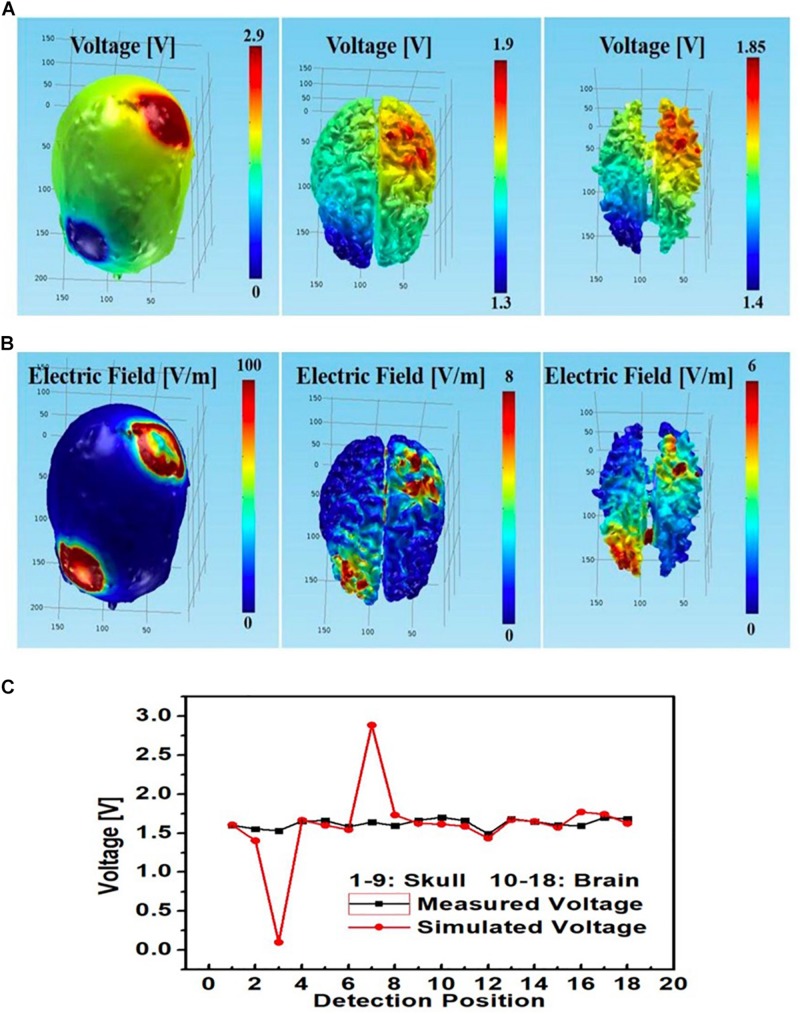
Left PMC anode and right supraorbital cathode montage using circular star array electrodes for stimulation; simulated **(A)** electric potential and **(B)** electric field in skull (left), gray matter (middle) and white matter (right); **(C)** simulated and measured voltages at the nine monitoring electrodes in the skull and brain layers.

See Figure 11 for comparisons between measured and simulated voltages at the 18 monitoring electrodes (i.e., nine at each of the skull and gray matter layers) in the circular array left PMC anode-right supraorbital cathode bilateral PMC montage. Again, measured voltages agreed with simulated values; however, it is important to notice the simulated vs. measured voltage differences between monitoring electrodes at skull and brain tissue layers (respectively, circular electrodes No. 3 and 12 cathodal, and No. 7 and 16 anodal), where simulated voltages at the skull had a sharp drop at the cathode (No. 3) and a sharp spike at the anode (No. 7), whereas measured voltages were steady at those locations on the skull; both measured and simulated voltages were similar in the brain layer under the anode and cathode (No. 12 and 16, respectively).

**FIGURE 12:**
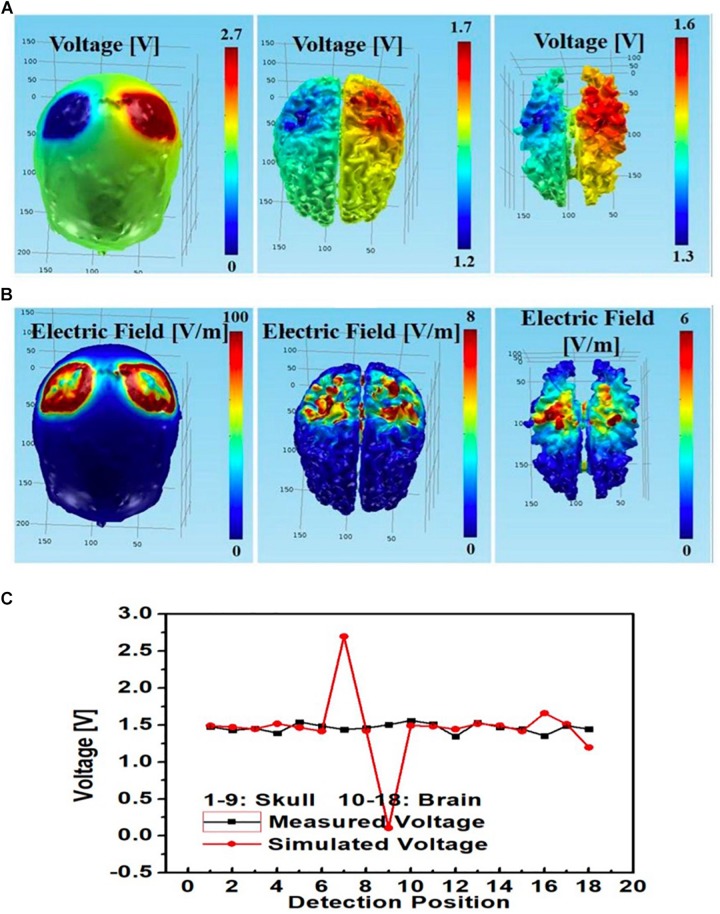
Bilateral PMC montage with left PMC anode and right PMC cathode using circular star array electrodes for stimulation; simulated **(A)** electric potential and **(B)** electric field in skull (left), gray matter (middle) and white matter (right); **(C)** simulated and measured voltages at the 9 monitoring electrodes in the skull and brain layers.

An additional tDCS stimulation montage with left PMC anode, right PMC cathode was also modeled. The simulated and measured voltages at different monitoring locations largely agreed at the brain layer; however, whereas measured voltages at the skull layer remained steady, the simulated voltage had a sharp spike at the left PMC anode (No. 7) and a sharp drop at right PMC cathode (No. 9) (Figure 12C).

**FIGURE 13:**
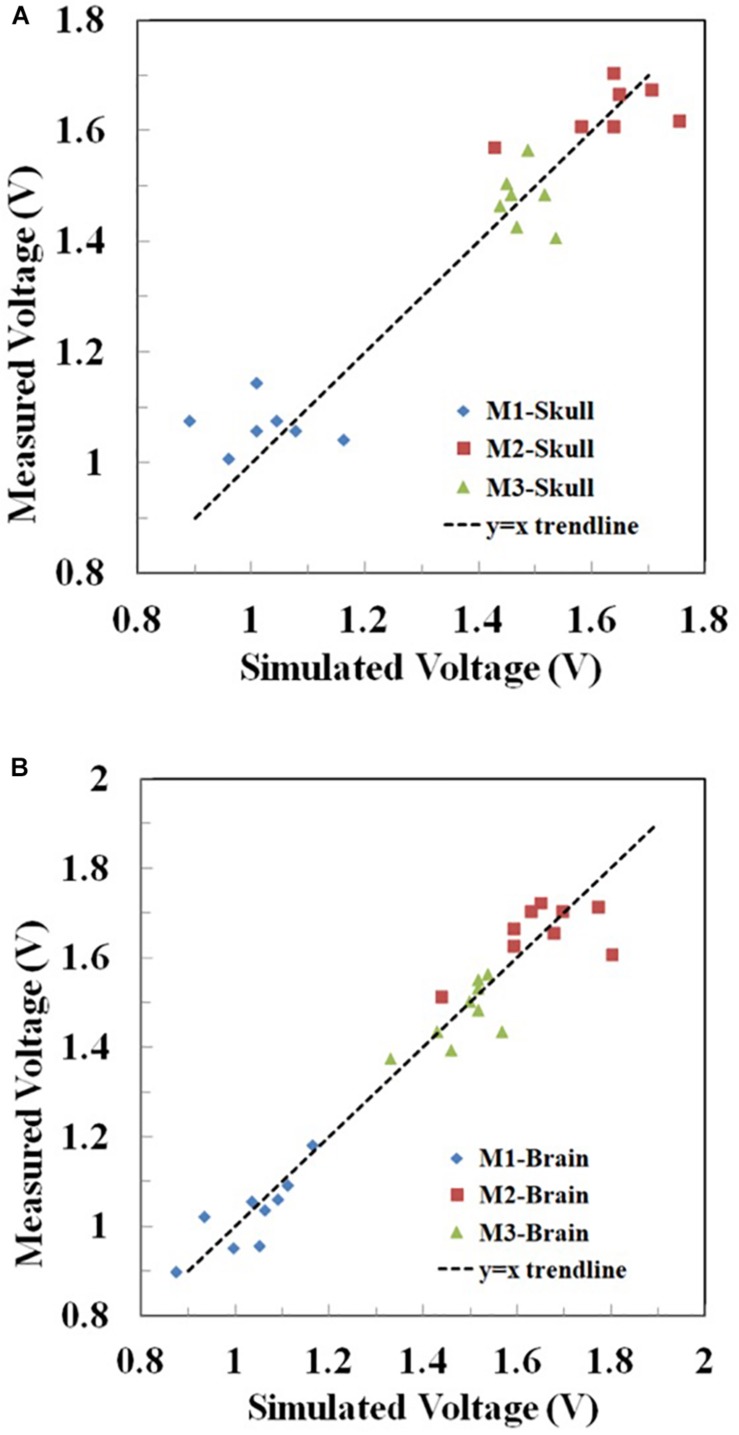
Correlation between the measured and simulated voltages in the **(A)** skull and **(B)** brain layer in Montages 1–3 (M1-3) reflecting the montages in Figures 10–12, respectively. The voltages at the two excitation electrodes were excluded for data analysis. For visual clarity, the *y* = *x* trendline was added in each figure.

Furthermore, for the rectangular electrode bilateral DLPFC montage (Figure 10) as well as the circular electrode left PMC-right supraorbital (Figure 11) and bilateral PMC montages (Figure 12) the correlations between simulated and measured voltages as quantified by the Pearson correlation coefficient were better in the brain layer (85, 79, and 88%, respectively) than the skull layer (69, 65, and 63%, respectively) (Figure 13).

**FIGURE 14:**
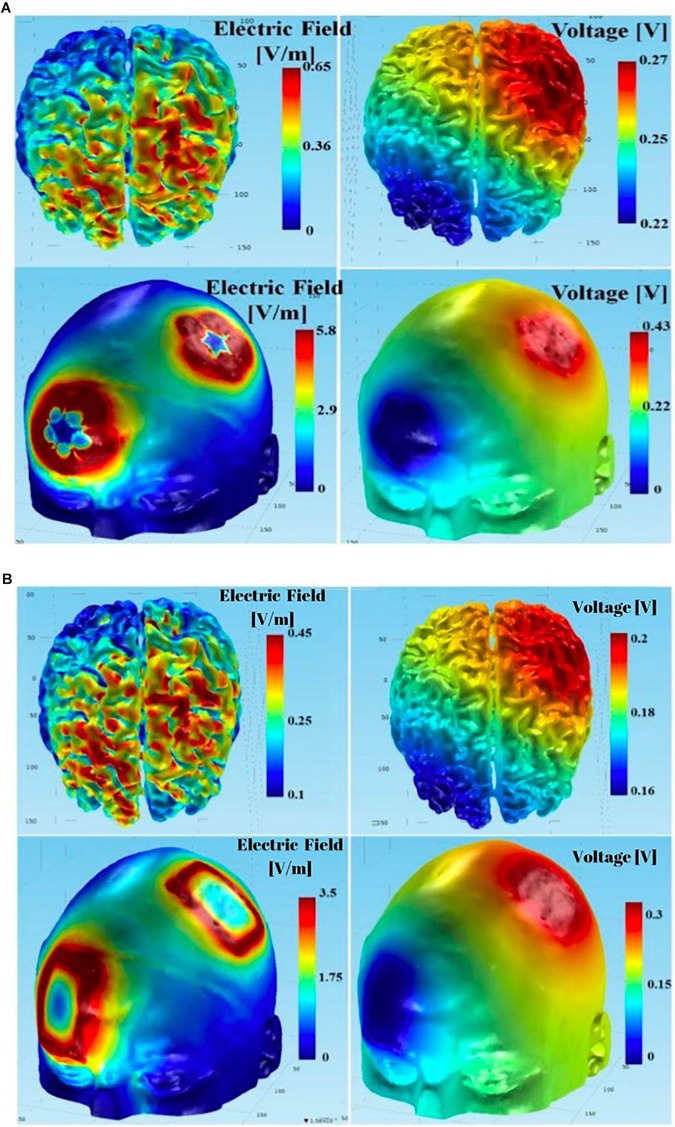
Using the complete model (including CSF in the simulations): **(A)** results of tDCS with anodal PMC - return at right supraorbital region montage using the circular array electrodes; **(B)** results of tDCS with anodal PMC - return at right supraorbital region montage using the rectangular sponge electrodes.

Additionally, the maximum electric fields in the gray and white matter tissue layers were slightly larger with the circular electrodes. Figure 14 summarizes the physics-based modeling (which included CSF modeling) results at the skull and brain tissue layers using circular star arrays (Figure 14A) and rectangular pad stimulation electrodes (Figure 14B) with a left PMC anode-right supraorbital cathode montage. Both simulations yielded similar results in that the electric field and voltage drop were larger at the stimulation locations. The only noticeable differences include the following two aspects: (a) modeled stimulation with the circular electrode array appeared to be more focused – specifically, the voltage distribution on the skull appeared to be more centered around the intended stimulation areas; and (b) the maximum electric field and potential drop in the gray matter and white matter tissue layers were larger using the circular array electrodes compared to the rectangular sponge electrodes. Both the electric field and potential drop were around 50% greater than those using the rectangular sponge electrode pair.

**FIGURE 15:**
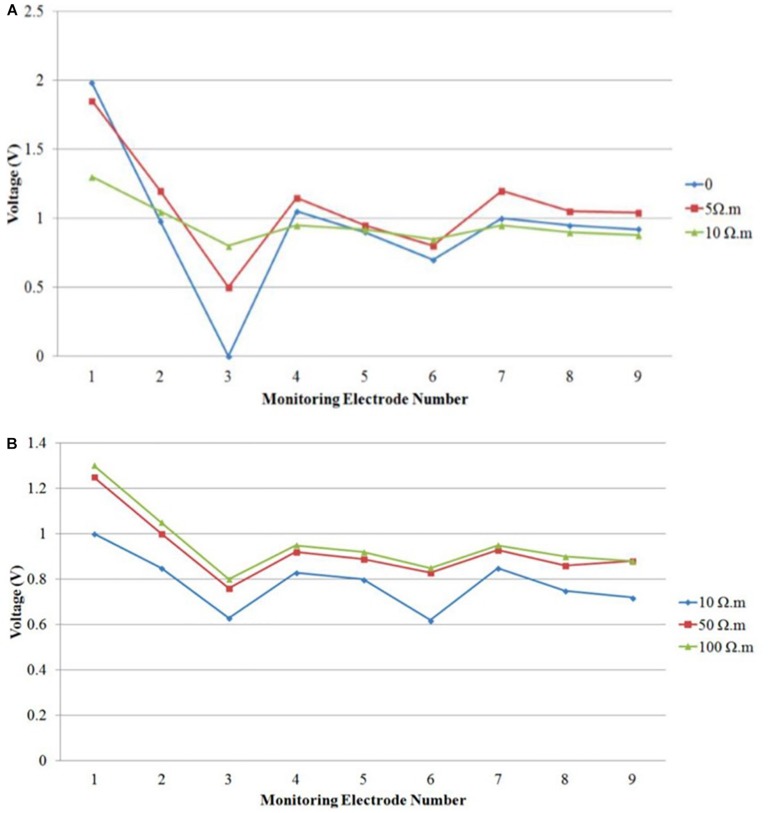
Simulated voltage distribution on the skull layer at nine monitoring electrodes using tDCS montage: anode over left DLPFC, cathode over right DLPFC, and using different contact gel resistivity **(A)** 0, 5, and 10 Ωm; **(B)** 10, 50, and 100 Ωm.

In order to identify the cause for the observed discrepancies between the simulated and measured voltages, particularly at the skull layer, we performed additional simulations by varying the contact resistances between the electrodes and skull. In the simulations, the contacts between the electrodes and the skull layer were assumed to be a perfect conductor with negligible resistivity. Figure 15A summarizes the simulation results at the skull layer for the rectangular electrodes in the bilateral DLPFC montage by varying the gel contact resistance from ideal to 10 Ωm. It showed that the voltages at the skull layer, particularly at the stimulation electrodes were largely affected by the contact resistivity. The voltage drop between the anode and cathode was ∼2 V for an ideal contact, which was much larger than the minor drop for a gel contact resistivity of 10 Ωm. Additionally, the voltage variations among different monitoring electrodes were much smaller with a larger gel contact resistivity. It should be noted that the voltages at the skull layers with a gel contact resistivity of 10 Ωm were very close to the actual measured results as shown in Figure 10C (left) for the same bilateral DLPFC montage. These results suggest that the contact resistance between the electrodes and the skull layer needs to be considered so that a realistic comparison between the theoretical modeling and experimental measurements can be achieved. In addition to the contact resistance, actual tissue resistivity difference may also be the cause of the observed discrepancy. Figure 15B shows the simulated voltages for the same montage at the nine monitoring electrodes in the skull layer with different skull resistivity (10, 50, and 100 Ωm). The gel contact resistivity of 10–100 Ωm was used in the simulations. Although the voltage drop between the cathode and anode was virtually unaffected, the absolute values of the voltages at the monitoring electrodes were dependent on the skull conductivity; the voltages were systematically higher at all monitoring electrodes for higher skull resistivity. Therefore, these results suggest that additional measurements are necessary to ensure that actual resistivity of the agar tissue simulants are close to those used in the physics-based modeling for efficient tDCS model validations.

## Discussion

This study applied electrical currents to a high-fidelity 3D head phantom model with monitoring electrodes embedded at the skull and brain (gray matter) compartments. While anatomical, physiological and functional variables modify tDCS stimulation outcomes, we investigated the effects of current delivery to different “anatomical” compartments in a physical model. The analysis compared the electrical currents injected into the phantom with those simulated in a physics-based model. These simulation results showed that it is advantageous to use the circular electrodes to achieve more focused stimulations in the desired brain areas. The results of our study show that the physical model is feasible and agrees with the computer simulations.

Although both voltage and electric field can be simulated, only the former quantity can be directly and reliably measured by the embedded monitoring electrodes. To determine the electric field at different monitoring locations, one would have to make certain assumptions regarding the effective electrical conducting path length based on the measured voltage data. Due to the complex shape and configuration of different tissues, it is difficult to determine the effective electrical conducting path length. Therefore, we used voltage measurements as they can be more reliably used to compare the experimental and simulated data for the purpose of tDCS modeling.

Physics-based modeling assumes good electrical contacts between the stimulation electrodes and skull surface, perhaps inaccurately. Our first measurement showed significant discrepancy between the stimulated and simulated models for monitoring electrodes No. 1 and 3 at the skull, under the rectangular anode and cathode, respectively. Because the contact resistances between electrodes and skull were in series connection with the phantom, poor electric contacts can lead to large potential drops in the contacts instead of the phantom brain. Consequently, a relatively large (i.e., ∼2 V) potential drop between the excitation anode and cathode was observed, measured by relative rather than absolute amplitudes. Current tDCS safety guidelines considered adequate electrode contact as important criteria for DC brain stimulation protocols (Bikson et al., 2016). In the bilateral DLPFC montage, our measurements of the electric potential and field distributions in gray matter agreed with previous bipolar simulations using computational modeling (Santos et al., 2016). Moreover, our phantom measurements followed the potential and field decay assumed within tissue depth.

Similarly, electric potential and field distributions in the left PMC-right supraorbital montage showed higher measurements at the skull surface, and lower potential and distribution in gray and white matter. However, compared to rectangular sponges, circular electrodes delivered a larger electric field over the gray matter and, to some extent, the white matter. We can therefore assume that circular electrodes in that montage increased current delivery in the phantom, which is important to consider when designing future therapeutic tDCS studies. TDCS precision targeting can be enhanced by changing electrode configurations and physical characteristics (Guler et al., 2016). Our phantom model detected differences resulting from stimulation electrode features, proving its sensitivity and functionality for future electrode arrays and to evaluate different electro-tissue interfaces. Overall, the measured and simulated voltages obtained for the left PMC-right supraorbital montage are similar to those in the literature (Bikson et al., 2012), including those in other validation models such as scalp voltage characterization (Datta et al., 2013). That said, it is possible that the measurement electrodes (being made of different materials) might have affected electric conductivity, or the electric field/current distribution. This limitation is similar to that of other models as well as *in vivo* recordings. Additionally, the white matter phantom with the plastic shell component may affect the volume conduction configuration in the adjacent gray matter compartment where we measured voltage changes. Our results also suggest that the actual resistivity of the agar should be measured to ensure that it is close to that of the physics-based model.

It is important to note that CSF was only modeled in Figure 14 (a more complete model than the simple 4-layer model in Figures 10–12), which shows that incorporating the CSF layer in the phantom model can affect the electric field magnitude dramatically (i.e., almost an order of magnitude difference). However, we intentionally omitted CSF; while adding the CSF tissue layer in the COMSOL multi-physics model for simulations would not cause much difficulty, replicating it with an agar phantom with our construction scheme (i.e., subsequently embedding one agar tissue in others) is very challenging. We instead used a simple 4-layer phantom to minimize the errors caused by discrepancies in the simulation model and the agar phantom. Utilizing this simpler phantom may affect the results in terms of spatial distribution and magnitude of the electric fields if one wants to predict those parameters in actual human brains based on the electrical measurements from the phantom. However, the current research was aimed at comparing physics-based modeling to a head phantom with structures and electrical properties which can closely mimic actual tissues. It should be emphasized that such a simple 4-layer phantom is just a first-step, preliminary structure. Complex phantoms with more tissue compartments should better predict the spatial distribution and magnitude of electric fields in actual human brains under different tDCS montages and conditions.

While all artificial models have limitations, such as those above and the one-month shelf-life for our phantom, yet this study approximates some of the anatomical properties involved in tDCS. The merits of the implemented head phantom and its fabrication method include the following:

(1) High accuracy for tDCS model physical optimization: the 3D printed head phantom can mimic both the physical structures and electrical conductivity distributions of various tissues, thus leading to very high accuracy for tDCS electrical modeling;

(2) Fully automatic process for tDCS parameter optimization: with the proposed reconfigurable DAQ, the stimulation montage and monitoring electrodes can be automatically varied to reveal current distribution in different brain areas. Such a fully automatic process allows the user to quickly optimize tDCS therapeutic treatment parameters;

(3) Capable of generating both generic and subject-specific phantoms: the 3D printing technique allows the fabrication of both generic and subject-specific phantoms. Therefore, the dimensional and structural differences among individuals can be considered to customize tDCS stimulation conditions for different individuals (including models with anatomical variations or implanted materials);

(4) Low fabrication cost: A digital fabrication file or an MRI image stack is all that is needed for head phantom fabrication. Such a method does not require any special tooling, such as injection molding, which is generally used to fabricate complex structures. Therefore, the implemented method allows for significant time and cost savings for phantom fabrication. We estimate that each phantom can be produced at $200/unit, if fabricated at a small volume.

## Conclusion

Our high-fidelity 3D head phantom model was feasible, comparable to computer-based electrical simulations, and supports further studies validating such simulations as well as testing the reliability of this model. This process allows us to better understand tDCS effects on different tissues and locations in a way that can be tailored to specific individuals, improving our ability to customize tDCS treatments at relatively low cost. Future work should explore the role of anatomical variations (normal and abnormal), electrode arrays, and different techniques for brain stimulation as these are not addressed in this model.

## Data Availability Statement

The datasets generated for this study are available on request to the corresponding author.

## Author Contributions

LM-Q, PL, and FF designed and conducted the study. PL developed the phantom and responsible for the data collection. LM, ME-H, and PL analyzed and interpreted the data. LM-Q, ME-H, PL, and BC prepared the manuscript draft with important intellectual input from FF and RM. All authors reviewed the manuscript critically for important intellectual content and approved the final version.

## Conflict of Interest

PL was employed by the company Alphasense. The remaining authors declare that the research was conducted in the absence of any commercial or financial relationships that could be construed as a potential conflict of interest.

## Abbreviations

ADC: analog-to-digital converter
B1: background image 1
CSF: cerebrospinal fluid
DAC: data acquisition circuit
DAQ: data acquisition
DLPFC: dorsolateral prefrontal cortex
EEG: electroencephalogram
FEM: finite element model
GND: ground
GUI: graphical user interface
M1: brain tissue mask 1
MUX: multiplexer
PMC: primary motor cortex
tACS: transcranial alternating current stimulation
tDCS: transcranial direct current stimulation
