# Supplementary material for: Transcranial Direct Current Stimulation Optimization – From Physics-Based Computer Simulations to High-Fidelity Head Phantom Fabrication and Measurements
Source: Front Hum Neurosci. 2019 Oct 31;13:388. doi: 10.3389/fnhum.2019.00388 (PMC6837166; doi:10.3389/fnhum.2019.00388)
Supplement: Supplementary file 1 [file Data_Sheet_1.docx]

**SUPPLEMENTARY MATERIALS**

**Transcranial Direct Current Stimulation Optimization – From Physics-Based Computer Simulations to High-Fidelity Head Phantom Fabrication and Measurements**

**
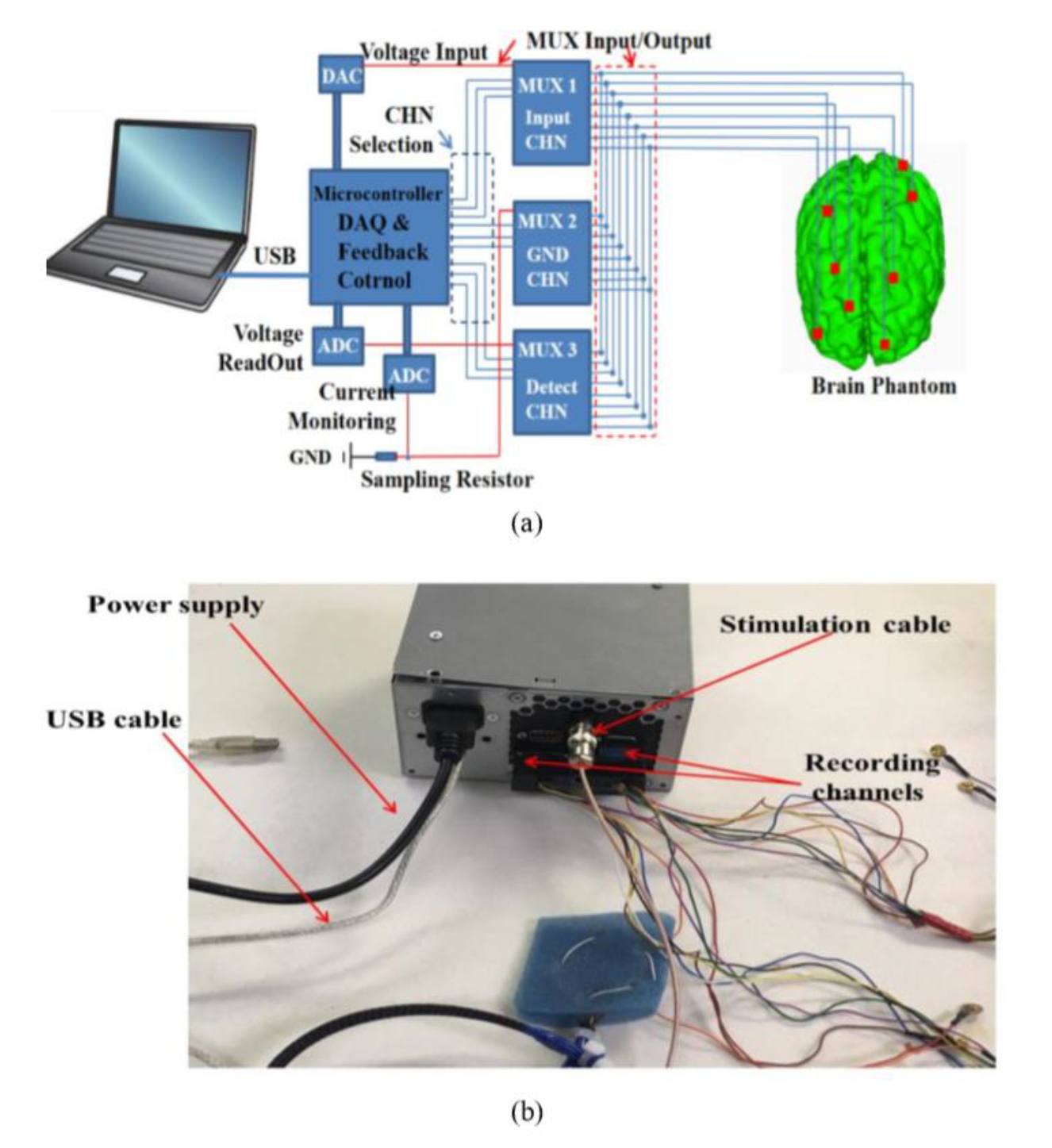
**

**Supplementary Figure 1 (a) A block diagram of the data acquisition circuit (DAC) for high- speed collections of electrical responses under different tDCS stimulation conditions, and (b) a picture of the implemented data acquisition device (DAQ).**

**
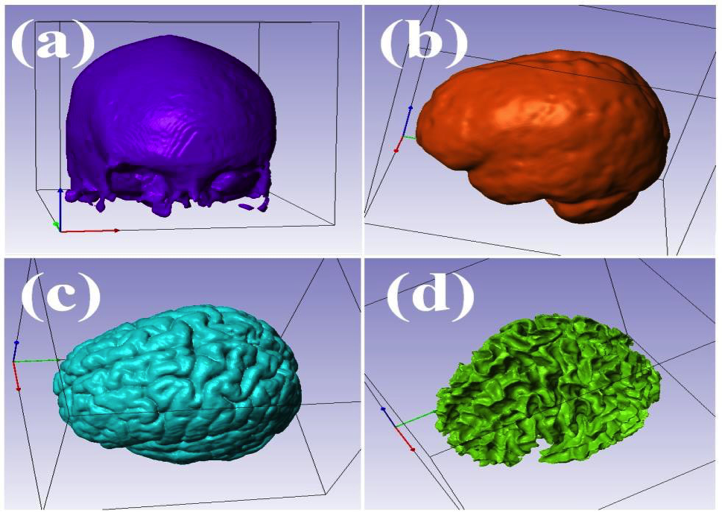
**

**Supplementary Figure 2: 3D tissue models for (a) skull, (b) CSF, (c) gray matter, and (d) white matter were extracted and processed from an MRI image stack.**

| **Tissue** | **Target Conductivity**  **(S/m)** | **Agar concentration**  **(g/l)** | **NaCl concentration**  **(g/l)** |
| --- | --- | --- | --- |
| **Scalp** | 0.465 | 30 | 2 |
| **Skull** | 0.01 | 30 | 0 |
| **Gray Matter** | 0.276 | 30 | 1.8 |
| **White Matter** | 0.126 | 30 | 0.5 |

**Supplementary Table 1: The conductivities and compositions of the electrical simulants for different tissues**
